# Baseline ASPECTS predicts early neurological deterioration and functional outcomes after endovascular thrombectomy in acute ischemic stroke

**DOI:** 10.3389/fneur.2025.1632149

**Published:** 2025-11-28

**Authors:** Wenke Tang, Yuqin Yi, Xia Peng, Zhenyong Xiao, Xianlei Yan, Hongmou Chen, Qidan Huang

**Affiliations:** Department of Neurosurgery, Liuzhou Worker's Hospital, Liuzhou, Guangxi, China

**Keywords:** early neurological deterioration, stroke, endovascular treatment, ASPECTS score, prognosis

## Abstract

**Background:**

Early neurological deterioration (END), defined as neurological decline after endovascular thrombectomy (EVT) for acute ischemic stroke (AIS), is a critical complication associated with poor long-term prognosis. Although END significantly impacts clinical outcomes, its underlying mechanisms and modifiable risk factors remain poorly understood. This study aimed to (1) characterize the clinical profile of END and (2) evaluate its association with 90-day functional outcomes in AIS patients who underwent EVT.

**Methods:**

This retrospective study included consecutive patients with acute ischemic stroke (AIS) secondary to large vessel occlusion (LVO) who were treated with endovascular thrombectomy (EVT) between January 2021 and December 2024. END was defined as either a ≥4-point increase in the National Institutes of Health Stroke Scale (NIHSS) score or a ≥1-point increase in the NIHSS consciousness subscore (Item Ia) within 72 h after EVT. Patients meeting these criteria were classified into the END group; others comprised the non-END group. Variables with a *p*-value of <0.05 in univariate analysis were included in a multivariable logistic regression model to identify independent predictors of END. The predictive performance of the Alberta Stroke Program Early CT Score (ASPECTS) was assessed through receiver operating characteristic (ROC) analysis, with the optimal cutoff determined by maximizing the Youden index.

**Results:**

The study included 177 consecutive AIS patients with LVO who were treated with EVT. END occurred in 52 patients (29.4%). The END group had significantly lower baseline ASPECTS values than the non-END group (median [IQR]: 7 [6–8] vs. 9 [8–9], *p* < 0.001). In the adjusted analysis, higher ASPECTS was independently associated with a reduced risk of END (OR = 0.59 per 1-point increase, 95% CI: 0.44–0.80, *p* = 0.001). ROC analysis identified an ASPECTS ≤7 as the optimal cutoff for predicting END (AUC = 0.761; sensitivity 75.0%, specificity 76.8%). At 90 days, functional independence [modified Rankin Scale (mRS) 0–2] was achieved in 52.0% of the non-END group versus 7.6% of the END group (*p* < 0.001).

**Conclusion:**

END after EVT independently predicts poor 90-day functional independence (mRS 0–2) in LVO-related AIS. Our findings support incorporating a baseline ASPECTS ≤7 into risk stratification protocols to identify high-risk patients requiring intensive neuromonitoring (hourly NIHSS assessments for 72 h post-EVT). Early detection of END signs, such as a ≥ 4-point increase in the NIHSS score 4, may enable prompt interventions (e.g., blood pressure control or edema management) to improve long-term outcomes.

## Introduction

Stroke remains the second leading cause of death globally when considering all causes and the third leading cause of death when categorized among specific diseases, as per the WHO. Acute ischemic stroke (AIS) accounts for approximately 62.4% of incident stroke cases (1). As the predominant stroke subtype, AIS imposes substantial socioeconomic burdens, necessitating continuous optimization of prevention and treatment strategies (1). Endovascular thrombectomy (EVT) has revolutionized the management of AIS caused by large vessel occlusion (LVO), demonstrating high recanalization rates and improved functional outcomes (2–4). However, early neurological deterioration (END)—defined as a ≥4-point increase in the National Institutes of Health Stroke Scale (NIHSS) score or ≥1-point increase in the NIHSS consciousness subscore (Item Ia) within 72 h post-EVT—has emerged as a critical complication, with contemporary studies reporting an incidence exceeding 18.8% (5–9). Imaging-based classifications categorize END into four subtypes: vasogenic edema (VE), ischemic progression (IP), symptomatic intracranial hemorrhage (sICH), and unexplained neurological decline (5–7). Despite its clinical significance, the pathophysiology of END remains poorly understood, particularly in the context of EVT. Early neurological deterioration (END) following EVT is multifactorial, involving mechanisms such as reperfusion injury, oxidative stress, inflammation, and blood–brain barrier (BBB) disruption. Key molecular pathways include upregulation of matrix metalloproteinases (MMPs)—particularly MMP-9—which degrade tight junction proteins, leading to vasogenic edema. Ischemic progression may involve excitotoxicity, calcium overload, and mitochondrial dysfunction. Hemorrhagic transformation is often mediated by reperfusion injury and endothelial damage. These processes collectively contribute to END and poor functional outcomes. The Alberta Stroke Program Early CT Score (ASPECTS), a validated neuroimaging tool for quantifying early ischemic changes, has proven valuable in guiding thrombolytic therapy by correlating with baseline NIHSS scores (10, 11). However, evidence regarding its role in predicting post-EVT END and its interactions with clinical variables remains limited.

This study aimed to investigate the association between baseline ASPECTS, clinical parameters, and END occurrence, as well as to assess the impact of END on 90-day functional independence [modified Rankin Scale (mRS) 0–2]. Our findings may refine risk stratification protocols and inform personalized therapeutic approaches for EVT-treated AIS patients.

## Methods

### Patient identification and selection

This study included consecutive patients with acute ischemic stroke (AIS) secondary to radiologically confirmed large vessel occlusion (LVO) who underwent endovascular thrombectomy (EVT) between January 2021 and December 2024. All participants underwent standardized neuroimaging, including pre- and post-procedural CT, CTA, MRA, or DSA, to confirm LVO and assess procedural success (a modified Thrombolysis in Cerebral Infarction score ≥2b).

The inclusion criteria were as follows: Age ≥18 years; radiologically confirmed LVO involving the intracranial internal carotid artery (ICA), middle cerebral artery (MCA) (M1 segment), or vertebrobasilar system (VA-BA); symptom onset-to-admission time ≤24 h; post-EVT follow-up neuroimaging within 72 h; and serial National Institutes of Health Stroke Scale (NIHSS) assessments documented at predefined intervals—immediately post-EVT, every 12 h for 72 h, and additionally upon any neurological deterioration (e.g., severe headache, altered consciousness, and vomiting).

The exclusion criteria were as follows: Incomplete baseline data due to in-hospital mortality before EVT; referral to external centers or contraindications to EVT (e.g., comorbidities, patient refusal); and loss to 90-day follow-up.

### Clinical parameters

Demographic and clinical variables were systematically collected, including baseline characteristics: Age (years) and sex; comorbidities: Hypertension, coronary artery disease, diabetes mellitus, and prior atrial fibrillation; stroke history: Previous ischemic or hemorrhagic events; physiological measures on admission: Systolic and diastolic blood pressure (SBP/DBP, mmHg) and fasting blood glucose levels (mmol/L); lifestyle factors: Current smoking status (defined as ≥1 cigarette/day in the past 6 months) and alcohol consumption (≥2 standard drinks/day); neuroimaging and procedural metrics: Baseline Alberta Stroke Program Early CT Score (ASPECTS) and NIHSS scores at admission and 24 h post-EVT; procedural timelines: Door-to-puncture time (min) and thrombectomy duration (min); and endpoint classification: END subtypes per imaging criteria, including vasogenic edema (VE), ischemic progression (IP), symptomatic intracranial hemorrhage (sICH), and indeterminate causes.

Functional outcomes were assessed at 90 days through blinded telephone interviews using the modified Rankin Scale (mRS), with evaluators masked to patient allocation and intraoperative findings.

The study was approved by the Ethics Committee of Liuzhou Workers’ Hospital.

## Outcome assessment

### Definition of END

Early neurological deterioration (END) was defined as either (1) an increase in the National Institutes of Health Stroke Scale (NIHSS) score by ≥4 points or (2) a ≥1-point increase in the NIHSS consciousness subscore (Item Ia) within 72 h following endovascular thrombectomy (EVT) (5, 12–15). Patients meeting these criteria were classified into the END cohort, while those without neurological decline comprised the non-END group. Procedural approaches adhered to contemporary guidelines (4), including first-line stent retriever thrombectomy (Class I-A recommendation), with adjunctive modalities, such as angioplasty, stent implantation, or bridging intravenous thrombolysis, employed based on intraoperative findings.

### END subtype classification

Post-procedural END etiologies were categorized using multimodal neuroimaging. Vasogenic edema (VE): Characterized by restricted diffusion on MRI-DWI (hyperintensity) with corresponding hypointensity on apparent diffusion coefficient (ADC) maps (16). Ischemic progression (IP): Radiological evidence of infarct expansion on serial CT or MRI, defined as a ≥30% increase in volume compared to baseline imaging (17, 18). Symptomatic intracranial hemorrhage (sICH): Parenchymal hematoma or intraventricular hemorrhage on CT with neurological deterioration (NIHSS increase ≥2) (19). Indeterminate END: Neurological decline without radiographic correlates after exhaustive evaluation (17).

### CT-ASPECTS evaluation

The Alberta Stroke Program Early CT Score (ASPECTS) is a validated 10-point neuroimaging grading system, initially proposed by Barber et al. (10) in 2000, for assessing early ischemic changes in anterior circulation stroke. The scoring protocol divides the middle cerebral artery (MCA) territory into 10 anatomical regions: M1–M6 cortical zones, the lentiform nucleus, caudate nucleus, internal capsule, and insular cortex. Each unaffected region is assigned 1 point, with a maximum score of 10. Hypoattenuation or parenchymal swelling within any designated region results in a 1-point deduction. For posterior circulation infarcts, the pc-ASPECTS scoring system, introduced by Puetz in 2008, evaluates 10 neuroanatomical structures across eight posterior circulation territories. Specifically: 1-point deduction: Unilateral thalamic, cerebellar hemispheric, or posterior cerebral artery (PCA) vascular territory involvement; 2-point deduction: Midbrain or pontine brainstem lesions. To minimize partial volume artifacts, hypodense lesions were required to be visually concordant across two consecutive axial CT slices. A normal ASPECTS/pc-ASPECTS value of 10 indicates the absence of ischemic injury, whereas a score of 0 denotes extensive infarction involving all assessed territories (20).

### Prognostic assessment

Functional independence was defined as a modified Rankin Scale (mRS) score of 0–2: mRS 0: No residual symptoms (asymptomatic); mRS 1: Minor symptoms without functional impairment; and mRS 2: Mild disability, able to perform self-care but unable to resume all pre-stroke activities. Scores of 3–6 indicated unfavorable outcomes: mRS 3: Moderate disability, requiring partial assistance for daily tasks (ambulatory without support); mRS 4: Severe disability, needing full assistance for mobility and self-care; mRS 5: Bedridden, completely dependent, and incontinent; and mRS 6: Mortality. To minimize bias, three independent teams were involved—imaging operators: Responsible for acquisition and interpretation of neuroimaging data, clinical therapists: Collected baseline data and post-procedural neurological assessments, and follow-up physicians: Performed blinded 90-day mRS evaluations via structured telephone interviews.

### Data analysis

The SPSS 26.0 software (IBM, Armonk, NY, USA) was used for all data analyses, and $\bar{x}$ ±s was used for normally distributed data. Student’s *t-*test was used for inter-group comparisons. Non-normal data were expressed as medians and quartiles [M (P25, P75)], and inter-group comparisons were performed using nonparametric rank-sum tests. Qualitative data were expressed as the number and percentage of cases [*n* (%)], and inter-group comparisons were performed using the *χ*^2^ test or Fisher’s exact test. Variables with statistical differences were further subjected to multivariate logistic regression analysis. If the *p*-value was < 0.05, the difference was considered statistically significant.

## Results

**Figure 1:**
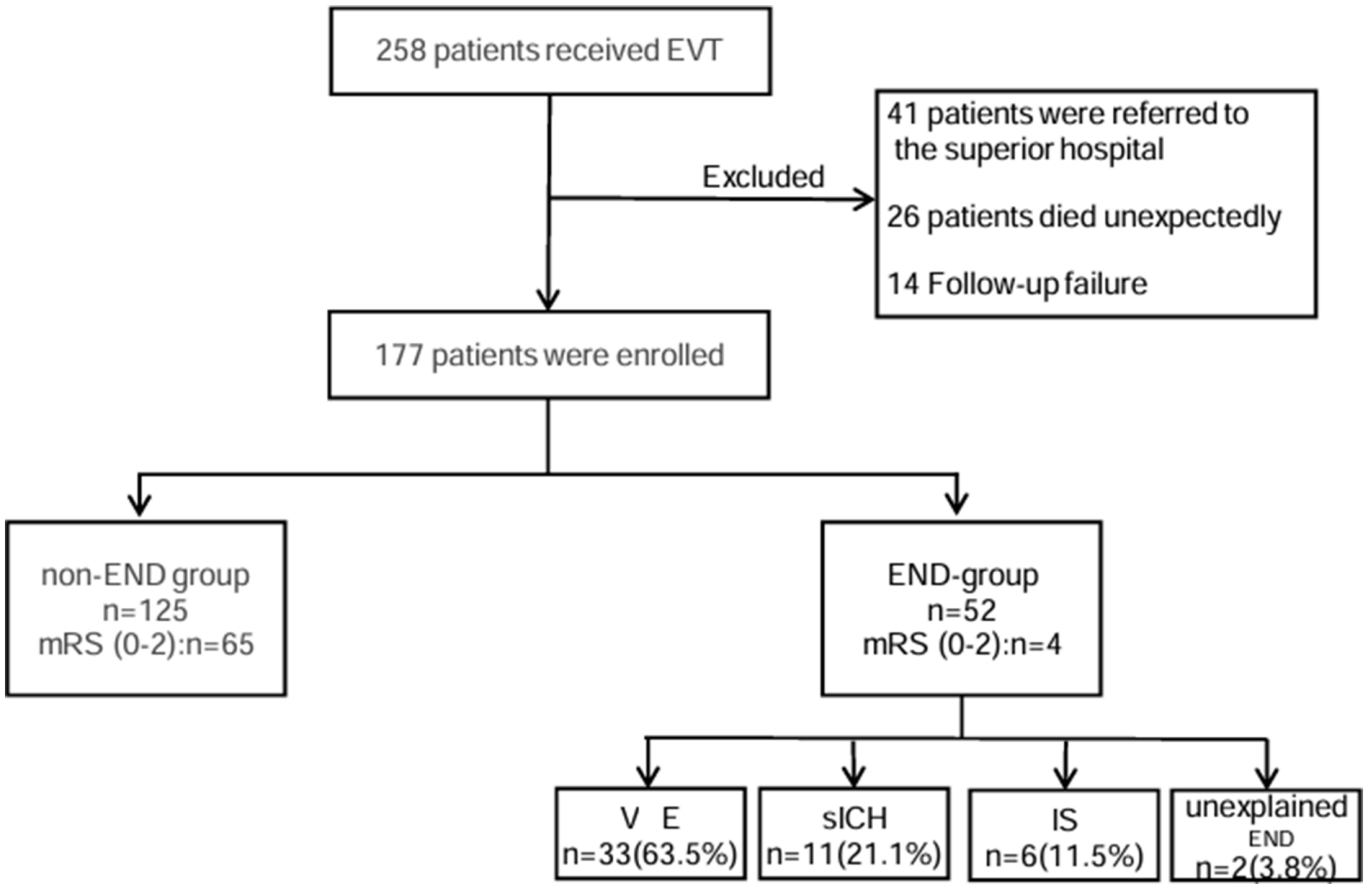
Flow diagram.

**Figure 2:**
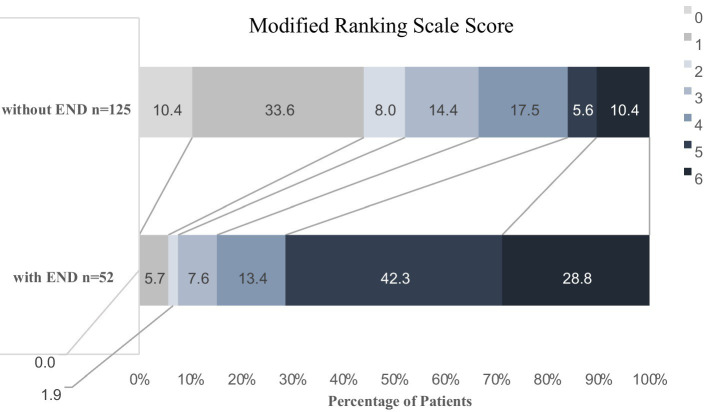
Modified Rankin Scale (mRS) score distribution between the END patients and non-END patients. The graph shows the percentage of patients in each group. MRS scores range from 0 (asymptomatic) to 6 (dead).

A total of 258 patients received EVT, and a total of 177 patients were ultimately included in our analysis. END occurred in 52 cases (29.4%). These included 33 (63.5%) cases of VE, 11 (21.1%) cases of sICH, six (11.5%) cases of IS, and two (3.8%) cases of unexplained END (Figure 1). In total, four patients (7.6%) in the END group achieved functional independence (mRS 0–2), and 65 patients (52%) in the non-END group achieved functional independence (mRS 0–2) (Figure 2).

### Baseline characteristics of the study cohort: patients with and without END

At the time of admission, the patients who developed END had higher SBP, elevated blood glucose levels, higher NIHSS scores, and a low early CT score for the Alberta Stroke Program. There was a significant difference in smoking history between the two groups (*p* < 0.001). Anterior circulation infarction was common in both groups, occurring in 107 patients (85.6%) in the non-END group and 41 patients (78.8%) in the END group. However, there was no significant difference in occlusion location between the two groups (*p* = 0.269). Demographic and other clinical characteristics of the two groups are presented in Table 1.

**Table 1:** Clinical features and related factors in the patients with and without END.

Variable	Non-END group	END group	*p*-value
Age, years	61.6 ± 10.8	61.9 ± 8.7	0.847
Male	94 (75.2%)	41 (78.8%)	0.603
Hypertension	65 (52%)	38 (73%)	**0.010**
Heart disease	22 (17.5%)	11 (21.1%)	0.580
Diabetes	30 (24%)	17 (32.6%)	0.233
Atrial Fibrillation	32 (25.6%)	12 (23%)	0.724
Smoking History	31 (24.8%)	20 (38.4%)	0.068
SBP, mmHg	159 ± 27	180 ± 34	**<0.001**
DBP, mmHg	95 (81, 108)	100 (86, 114)	0.157
Admission blood glucose, mmol/L	7.9 (6.9, 10.3)	9.0 (7.3, 12.6)	**0.041**
Drinking history	43 (34.4%)	23 (44.2%)	0.218
Preoperative CT-ASPECTS score	9 (8, 9)	7 (6, 8)	**<0.001**
Admission NIHSS score	16 (13, 20)	16 (16, 20)	0.344
Intravenous thrombolysis	40 (32%)	17 (32.6%)	0.928
From admission to EVT, min	120 (95, 151)	162 (120, 186)	**<0.001**
From the start to the end of EVT, min	100 (80, 120)	100 (90, 132)	0.231
Occlusion position			0.269
Anterior circulation stroke	107 (85.6%)	41 (78.8%)	
Posterior circulation stroke	18 (14.4%)	11 (21.1%)	
90-day follow-up mRS			**<0.001**
mRS 0–2	65 (52%)	4 (7.6%)	
mRS 3–6	60 (48%)	48 (92.3%)	

Remark: The value in bold indicates a *p*-value of < 0.05.

SBP, Systolic Blood Pressure; DBP, Diastolic Blood Pressure; NIHSS, National Institutes of Health Stroke Scale; EVT, Endovascular Treatment; CT-ASPECTS, CT Arterial Spin Labeling Perfusion Score; mRS, Modified Rankin Scale.

Data are presented as mean ± SD, *n* (%), or median (P25, P75).

### Intraventricular hemorrhage is an independent risk factor for the development of chronic hydrocephalus

Further multivariate logistics regression analysis included the following variables: History of hypertension, systolic blood pressure on admission, blood glucose on admission, smoking history, preoperative CT-ASPECTS value, and time from admission to EVT. Among these, preoperative CT-ASPECTS value, hypertension history, smoking history, and time from hospital admission to EVT initiation were good independent predictors (Table 2).

**Table 2:** Multivariable logistic regression analysis of factors associated with END.

Variable	OR	95% CI	*p*-value
Age (per year)	1.01	0.98–1.04	0.472
Sex (male)	1.10	0.47–2.58	0.831
Hypertension	3.20	1.20–8.55	**0.020**
Admission glucose	1.08	0.96–1.22	0.201
Smoking History	8.90	3.35–23.67	**<0.001**
Preoperative ASPECTS (per pt)	0.59	0.44–0.80	**0.001**
Admission NIHSS	1.03	0.97–1.10	0.346
Door-to-puncture time	1.014	1.003–1.024	**0.011**

Remark: The value in bold indicates a *p*-value of < 0.05.

SBP, Systolic Blood Pressure; EVT, Endovascular Treatment; CT-ASPECTS, CT Arterial Spin Labeling Perfusion Score.

### Predictive efficacy of endovascular treatment timing on prognosis

**Figure 3:**
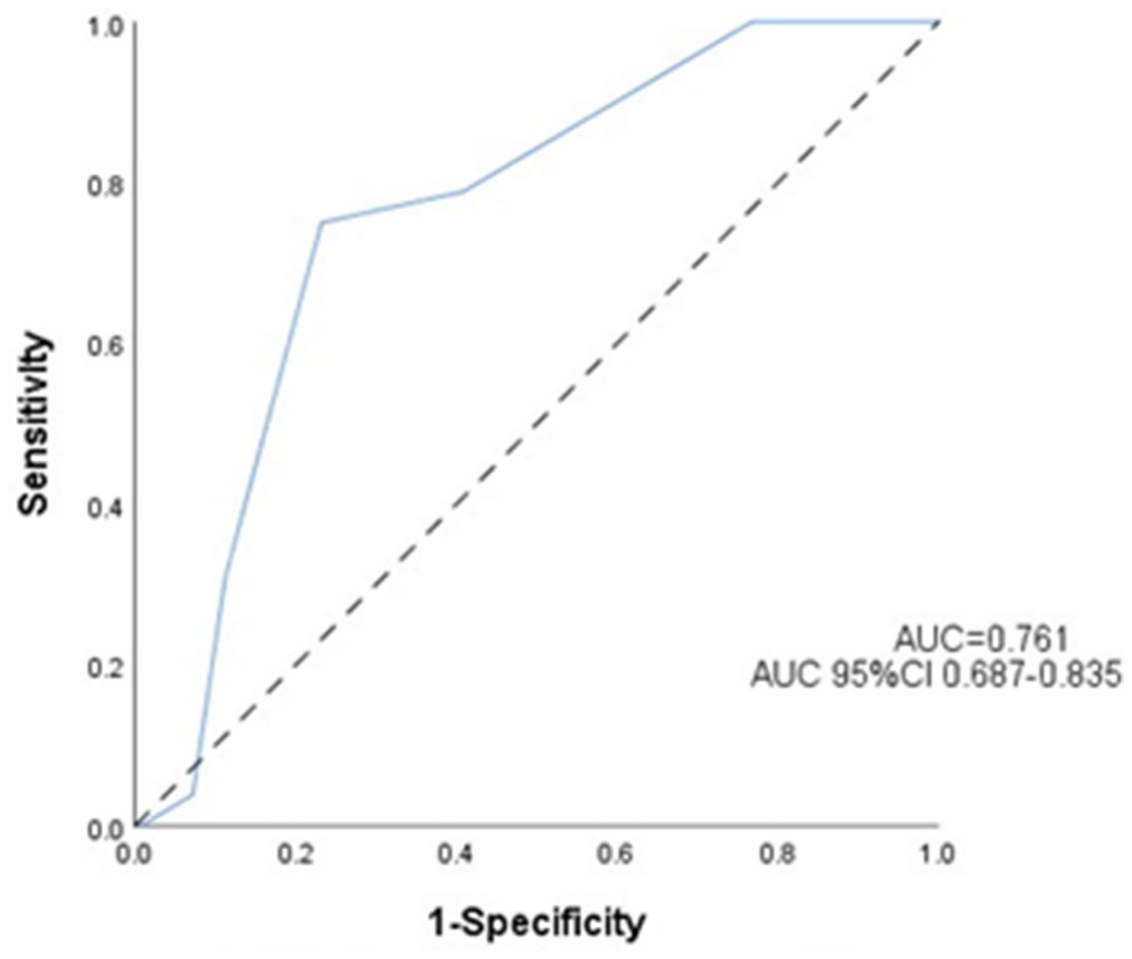
Receiver operating characteristic (ROC) curve for the prediction of early neurological deterioration. Predictive performance of the ASPECTS for early neurological deterioration.

Receiver operating characteristic (ROC) analysis is shown in Figure 3. ASPECTS values demonstrated good predictive value for END. The area under the curve was 0.761. An ASPECTS cutoff of ≤7 provided the optimal prediction, yielding a sensitivity of 75.0%, a specificity of 76.8%, and a 95% CI of [0.662–0.816].

## Discussion

In this cohort study, early neurological deterioration (END) occurred in 29.4% of the patients who underwent endovascular thrombectomy (EVT), with vasogenic edema (VE) representing the most prevalent subtype (63.5%, *n* = 33), followed by symptomatic intracranial hemorrhage (sICH; 21.1%, *n* = 11) and ischemic progression (IP; 11.5%, *n* = 8). These findings corroborate previous hypotheses that VE may emerge as the predominant END phenotype in the EVT era, potentially reflecting pathophysiological shifts in blood–brain barrier (BBB) integrity during reperfusion (5). Notably, the patients with VE exhibited significantly poorer functional outcomes compared to the sICH and IP subgroups, with only 3.0% achieving 90-day functional independence (mRS 0–2) versus 18.1 and 16.6%, respectively (*p* < 0.001). The heterogeneity in END incidence across studies likely stems from inconsistent diagnostic criteria and population variability, including differences in ischemia duration, collateral circulation status, and imaging protocols (5, 17). Our multivariable analysis identified four independent predictors of END: Chronic hypertension (OR = 3.2; 95% CI:1.20–8.55; *p* = 0.020); active smoking (OR = 8.9; 95% CI:3.35–23.67; *p* < 0.001); baseline ASPECTS ≤7 (OR = 0.59; 95% CI:0.44–0.80; *p* = 0.001); and prolonged door-to-puncture time (>162 min; OR = 1.014; 95% CI:1.003–1.024; *p* = 0.011). The predominance of VE may be attributable to BBB disruption during ischemia–reperfusion, facilitating cytotoxic edema through extravasation of sodium ions, plasma proteins, and inflammatory mediators—a cascade exacerbated by delayed EVT initiation. This mechanism aligns with experimental evidence demonstrating matrix metalloproteinase-9 (MMP-9)-mediated tight junction degradation within 6 h of ischemia.

Accumulating evidence underscores the prognostic utility of the baseline ASPECTS in acute ischemic stroke management. As demonstrated in previous studies (21), an ASPECTS value of ≤7 independently predicts unfavorable outcomes, with significantly higher rates of futile recanalization post-EVT compared to ASPECTS ≥7 cohorts (57.4% vs. 11.9%; *p* < 0.001). This threshold effect was further supported by MacCallum et al. (22), who identified an ASPECTS value ≤7 as a key predictor of malignant middle cerebral artery infarction. Our ROC analysis identified 7 as the optimal cutoff (AUC = 0.761; sensitivity 75.0%, specificity 76.8%; 95%CI:0.662–0.816), consistent with Tei et al. (23), who reported that an ASPECTS value ≥7 independently predicts favorable anterior circulation outcomes (mRS 0–2:44.9% vs. 29.4%; *p* = 0.002). The biological plausibility of this cutoff may relate to penumbral volume differentials: patients with an ASPECTS value of 8–10 retain substantial salvageable tissue (>70% ischemic penumbra) (24), enabling collateral recruitment and distal reperfusion post-EVT. Conversely, an ASPECTS value of 0–7 correlates with established infarct cores (>100 mL) (25), where impaired collateralization perpetuates ischemic cascades, ultimately exacerbating END risk (OR = 4.82; 95%CI: 3.11–6.94). Notably, while anterior circulation predominance was observed in the END cases (78.8% vs. 21.2%; *p* = 0.269), the ASPECTS demonstrated comparable predictive accuracy across vascular territories—Anterior circulation: AUC = 0.754 (75.6% sensitivity,75.7% specificity;95%CI: 0.673–0.805), and posterior circulation: AUC = 0.811 (72.7% sensitivity, 83.3% specificity;95%CI:0.640–0.981). Clinical Recommendations: patients with an ASPECTS ≤ 7, implementation of intensive neuromonitoring protocols and early initiation of neuroprotective therapies, such as therapeutic hypothermia.

Chronic hypertension is independently associated with early neurological deterioration (END) following endovascular thrombectomy (EVT). Contemporary evidence (24) highlights the dual challenges of acute blood pressure management in ischemic stroke and chronic hypertensive vasculopathy, wherein prolonged hypertension induces BBB disruption through aquaporin-4 (AQP4)-mediated edema formation. This pathophysiological cascade is exacerbated by oxidative stress resulting from nicotinamide adenine dinucleotide phosphate (NADPH) oxidase activation, which amplifies vascular permeability and early END susceptibility. A recent meta-analysis (25) revealed U-shaped associations between baseline systolic blood pressure (SBP) and post-thrombectomy outcomes, with optimal functional recovery (mRS 0–2) occurring at SBP 120–160 mmHg and mortality risk escalating beyond 177 mmHg. Although our study did not identify specific SBP thresholds for END prediction, chronic hypertension emerged as an independent predictor in the adjusted models (OR = 3.2; 95% CI: 1.20–8.55; *p* = 0.020), underscoring its value in preprocedural risk assessment.

Active smoking independently predicts early neurological deterioration (END) following endovascular thrombectomy (EVT), likely mediated through multifactorial pathways. Cigarette-derived toxins, including polycyclic aromatic hydrocarbons and reactive oxygen species, induce endothelial dysfunction via NF-κB-mediated inflammatory cascades, accelerating atherosclerosis and luminal stenosis (26). These prothrombotic alterations impair cerebral hemodynamics, reducing mean blood flow velocity by 15–20% in middle cerebral artery territories (*p* < 0.01) and thereby increasing ischemic penumbra vulnerability. Chronic smoking exacerbates hypoxic–ischemic injury through carboxyhemoglobin formation (CO-Hb > 5% in smokers vs. <1.5% in non-smokers), decreasing oxygen delivery capacity by 30–40% (27, 28). This oxygen debt potentiates cytotoxic edema, particularly in watershed zones, and may explain the predominance of vasogenic edema (VE) among smoking-related END cases (OR = 8.9; 95%CI: 3.35–23.67; *p* < 0.001). Mechanistically, nicotine upregulates matrix metalloproteinase-9 (MMP-9) expression by 2.5-fold compared to non-smokers, disrupting BBB integrity and facilitating VE pathogenesis post-EVT.

The pivotal DIRECT-MT trial (8) first showed that reducing door-to-puncture time (median 34 vs. 58 min) significantly decreases END incidence after endovascular thrombectomy (EVT) (17.3% vs. 28.9%; *p* = 0.02). Our findings corroborate this temporal relationship, demonstrating that each 30-min delay in EVT initiation increased END risk by 18% (HR = 1.18, 95%CI: 1.04–1.33; *p* = 0.016). While a meta-analysis (29) identified 7.3 h from symptom onset as the critical therapeutic window for maximum EVT benefit, our cohort’s frequent uncertainty regarding symptom onset times (42% unwitnessed strokes) precluded precise onset-to-treatment analysis—a limitation mitigated by prioritizing measurable in-hospital workflow metrics. These data underscore the imperative to streamline institutional stroke protocols, including implementing prehospital notification systems to accelerate CT-ASPECTS acquisition (<15 min from arrival), enabling parallel processing of neuroimaging interpretation and thrombectomy team activation, and establishing real-time quality-metric monitoring of door-to-puncture intervals (30). Notably, the ASPECTS demonstrated 89% interrater reliability in our emergency setting, enabling rapid triage without delaying reperfusion. For unwitnessed strokes, perfusion-diffusion mismatch analysis might extend EVT eligibility beyond traditional time windows—an approach that requires validation in future studies.

## Limitations

This study has several limitations that warrant consideration. As with all retrospective analyses, inherent biases, such as potential selection bias and unmeasured confounding variables, may influence the results. Therefore, our findings require validation through prospective, multicenter trials to address these inherent limitations. Regarding unmeasured therapeutic confounders, post-EVT interventions (e.g., decompressive craniectomy, targeted temperature management) were not analyzed for their prognostic impact. There was temporal sampling restriction, where END cases that developed beyond 72 h were excluded, potentially underestimating delayed deterioration mechanisms. Information on pre-procedure anticoagulation/antiplatelet use was not systematically collected in our registry-based study, which we acknowledge as a limitation.

## Conclusion

END after EVT was associated with poor 90-day functional independence. The Alberta Stroke Program Early CT Score (ASPECTS) emerged as a robust independent predictor of early neurological deterioration (END) following successful recanalization in acute ischemic stroke, with a preoperative ASPECTS value ≤7 demonstrating particular clinical utility. Multivariable analysis identified other modifiable risk factors significantly associated with END—chronic hypertension, smoking history, and prolonged door-to-puncture time. It can be seen that post-onset blood pressure management and shortening the time from onset to puncture play important roles in reducing early functional deterioration.

## Data Availability

The raw data supporting the conclusions of this article will be made available by the authors without undue reservation.
